# Major Potential Confounder Not Addressed

**DOI:** 10.1371/journal.pmed.0030063

**Published:** 2006-01-31

**Authors:** Jennifer Vines

**Affiliations:** **1**Oregon Health and Science UniversityPortland, OregonUnited States of America

*This is the first of 12 Correspondence pieces in this issue that are in response to an important PLoS Medicine Research Article: Auvert B, Taljaard D, Lagarde E, Sobngwi-Tambekou J, Sitta R, et al. (2005) Randomized, controlled intervention trial of male circumcision for reduction of HIV infection risk: The ANRS 1265 trial. PLoS Med 2(11): e298. Readers wishing to add their own views may do so using our E-letters facility, where the debate continues.*

In the article by Auvert et al. regarding incidence rates of HIV infection in circumcised versus uncircumcised men, the finding of 60% fewer infections among the former group is compelling [ 1]. I must echo the comments submitted by others and question these findings in light of the fact that the authors did not control for other sources of HIV transmission, such as exposure through blood transfusions or infected needles. While the literature supports sexual (primarily heterosexual) activity as the main route of HIV transmission in South Africa, the behavioral factor of “attending a clinic for a health problem related to the genitals,” initially reported by approximately 10% of both the intervention and the control group, corresponds to a significantly elevated HIV incidence rate. It is plausible that these men presented with urogenital complaints that resulted in antibiotic or other therapeutic treatments administered with unsterile needles. This could represent a significant confounder since the uncircumcised men, if indeed more prone to sexually transmitted infections (STI), were more likely to present for STI care and become infected through the health-care setting rather than through unprotected sexual intercourse. Controlling for this route of infection could result in a smaller difference between HIV infection rates in the circumcised versus uncircumcised groups, indicating that circumcision may not be as effective at decreasing HIV transmission as the article suggests.
